# The association between maternal obesity and fetomaternal outcomes in twin pregnancies

**DOI:** 10.1371/journal.pone.0306877

**Published:** 2024-07-10

**Authors:** Leandra Nagler, Carmen Eißmann, Marita Wasenitz, Franz Bahlmann, Ammar Al Naimi

**Affiliations:** 1 Goethe University Frankfurt am Main, Frankfurt, Hessen, Germany; 2 Department of Obstetrics and Gynecology, Buergerhospital ‐ Dr. Senckenbergische Stiftung, Frankfurt am Main, Hessen, Germany; 3 Department of Obstetrics and Prenatal Medicine, University Hospital, Goethe University Frankfurt, Frankfurt am Main, Hessen, Germany; University of Illinois Medical Center at Chicago: University of Illinois Hospital, UNITED STATES OF AMERICA

## Abstract

The prevalence of overweight and obese people worldwide has dramatically increased in the last decades and is yet to peak. At the same time and partly due to obesity and associated assisted reproduction, twinning rates showed a clear rise in the last years. Adverse fetomaternal outcomes are known to occur in singleton and twin pregnancies in overweight and obese women. However, the impact of the obesity levels as defined by the World Health Organization on the outcomes of twin pregnancies has not been thoroughly studied. Therefore, the purpose of this study is to examine how maternal overweight, and the level of obesity affect fetomaternal outcomes in twin pregnancies, hypothesizing a higher likelihood for adverse outcomes with overweight and each obesity level. This is a retrospective cohort study with 2,349 twin pregnancies that delivered at the Buergerhospital Frankfurt, Germany between 2005 and 2020. The mothers were divided into exposure groups depending on their pre-gestational body mass index; these were normal weight (reference group), overweight and obesity levels I, II, and III. A multivariate logistic regression analysis was performed to assess the influence of overweight and obesity on gestational diabetes mellitus, preeclampsia, postpartum hemorrhage, intrauterine fetal death, and a five-minutes Apgar score below seven. The adjusted odds ratio for gestational diabetes compared to normal weight mothers were 1.47, 2.79, 4.05, and 6.40 for overweight and obesity levels I, II and III respectively (*p* = 0.015 for overweight and *p* < 0.001 for each obesity level). Maternal BMI had a significant association with the risk of preeclampsia (OR 1.04, *p* = 0.028). Overweight and obesity did not affect the odds of postpartum hemorrhage, fetal demise, or a low Apgar score. While maternal overweight and obesity did not influence the fetal outcomes in twin pregnancies, they significantly increased the risk of gestational diabetes and preeclampsia, and that risk is incremental with increasing level of obesity.

## Introduction

Twinning rates were subject to many changes worldwide in the past few decades [1]. With a global increase of twin deliveries to 42%, a new peak has been reached between 2010 and 2015 [2]. Studies show that this development is mainly due to an increased use of assisted reproductive techniques (ART) [2], as well as increasing maternal age [3], the latter being accompanied by a higher likelihood for multiple pregnancies [4]. Both these trends can also be observed in Germany, where twinning rates increased by 29% and maternal age by 4.8% between 2005 and 2015 [5].

According to the World Obesity Federation [6], the prevalence of obesity (body mass index (BMI) ≥ 30 kg/m^2^) is globally expected to increase from 14% to 24% in only fifteen years until 2035. In this same period, the number of obese women aged over twenty years will increase by almost 10%. Considering the negative impact of obesity on female fertility [7], maternal obesity favoring twin pregnancies [8], and a subsequent increased use of ART in combination with rising maternal age, increasing twinning rates are therefore no surprise.

In singleton pregnancies, overweight and obesity lead to an 18–47% higher risk for obstetric complications [9]. The impact of overweight and obesity on fetomaternal outcomes has been thoroughly investigated in twin pregnancies as well, showing higher risks for gestational diabetes mellitus (GDM), gestational hypertension, preterm birth, or cesarean sections among many others [10]. However, the influence of the different obesity levels as defined by the Word Health Organization still lacks research. Thus, this study aims to examine the impact of overweight and the obesity levels on fetomaternal complications and outcomes in twin pregnancies, hypothesizing that the higher the maternal weight and the obesity level, the higher the likelihood for adverse outcomes.

## Materials and methods

This is a retrospective cohort study of 2,349 pregnant women delivering twins between 1^st^ of January 2005 and 31^st^ of December 2020 at the Buergerhospital in Frankfurt, Germany. The exposure of the mothers was classified according to their pre-pregnancy BMI (calculated as weight [kg] divided by the square of height [m^2^]) based on World Health Organization standards (normal weight 18.5–24.9 kg/m^2^, overweight 25–29.9 kg/m^2^, obesity level I 30–34.9 kg/m^2^, obesity level II 35–39.9 kg/m^2^ and obesity level III ≥ 40 kg/m^2^) [11]. Women of normal weight were used as a reference group.

Examined maternal outcomes included GDM, preeclampsia and postpartum hemorrhage (PPH), whereas the analyzed neonatal outcomes were fetal demise of at least one twin and an Apgar score below seven after five minutes. GDM was diagnosed according to the guidelines of the German Society of Gynecology and Obstetrics [12]. Every gravida underwent a 75 g oral glucose tolerance test between 24 and 28 weeks of gestation. Preeclampsia was defined according to the Practice Bulletin of the American College of Obstetricians and Gynecologists [13] as a blood pressure of ≥ 140/90 mmHg and proteinuria or at least one sign of organ damage. PPH was defined as bleeding > 1,000 ml or blood loss with signs of hypovolemia within twenty-four hours after birth. Intrauterine fetal death was defined according to the Royal College of Obstetricians & Gynaecologists as missing signs of life in utero after 24 completed weeks of gestation [14].

Univariate and multivariate logistic regression was utilized to assess the crude and adjusted odds ratios for our binomial outcomes. Differences in baseline characteristics between the exposure groups were reported in frequencies and proportions, means and standard deviation as well as medians and interquartile ranges. The reported *p*-values of differences were acquired with anova and chi-square test. The following covariates: age, use of ART, primigravida, chorionicity, gestational age, history of cesarean sections and abortions, smoking status, and pre-existing hypertension as well as diabetes mellitus type I or II were adjusted for in the multivariate regression, as they represented confounders for all examined outcomes and were obesity related. Gestational diabetes mellitus was not adjusted for the covariate pre-existing diabetes mellitus type I or II, and only the outcomes PPH, fetal demise and Apgar score were adjusted for gestational age. Chorionicity was included as a confounder in the adjusted analysis instead of being used for stratification. This decision was met to reduce the number of tables needed for a stratified analysis. Furthermore, the association of BMI as a continuous exposure with all outcomes was tested. A *p*-value < 0.05 was considered statistically significant, and all statistical analyses were conducted using Stata® (ver. 18, Texas, USA). This study has been approved by the ethics committee of the Medical Association of Hesse (January 3^rd^, 2022, reference number: 2021-2675-evBO). The need for patient’s consent was waived by the ethics committee and all data were fully anonymized. All authors had access to information that could identify individual participants during and after data collection. The data were accessed on February 7^th^, 2022, for research purposes.

## Results

A total of 2,349 mothers and their offsprings were analyzed. Baseline characteristics of the mothers and the descriptive statistics are shown in Table 1.

**Table 1:** Maternal characteristics.

Characteristics	Normal weight (n = 1,572)	Over-weight (n = 512)	Obesity level I (n = 171)	Obesity level II (n = 58)	Obesity level III (n = 36)	*p*-Value	
	n (%)	n (%)	n (%)	n (%)	n (%)	Normal weight vs. overweight and all levels	Obesity level I vs. II vs. III
Age^a^	33.8 (5.0)	33.6 (5.2)	32.8 (5.1)	31.8 (5.8)	32.1 (4.8)	0.001	0.430
BMI^b^	21.7 (20.3–23.0)	26.7 (25.7–28.0)	32.0 (30.9–33.4)	37.0 (36.2–38.1)	43.2 (41.7–44.3)	< 0.001	< 0.001
ART	589 (37.5%)	178 (34.8%)	50 (29.2%)	14 (24.1%)	12 (33.3%)	0.064	0.600
Gestational age (in weeks)						0.620	0.520
<24	11 (0.7%)	7 (1.4%)	3 (1.8%)	1 (1.7%)	0 (0%)		
24–27+6	45 (2.9%)	18 (3.5%)	3 (1.8%)	3 (5.2%)	2 (5.6%)		
28–31+6	97 (6.2%)	30 (5.9%)	13 (7.6%)	6 (10.3%)	1 (2.8%)		
32–36+6	719 (45.7%)	230 (44.9%)	68 (39.8%)	24 (41.4%)	20 (55.6%)		
≥ 37+0	700 (44.5%)	227 (44.3%)	84 (49.1%)	24 (41.4%)	13 (36.1%)		
Primigravida	801 (51.0%)	215 (42.0%)	64 (37.4%)	23 (39.7%)	8 (22.2%)	< 0.001	0.170
Gravidity^b^	1.0 (1.0–2.0)	2.0 (1.0–3.0)	2.0 (1.0–3.0)	2.0 (1.0–3.0)	2.0 (2.0–3.5)	< 0.001	0.160
Dichorionic twins	1,220 (78.0%)	387 (76.0%)	135 (79.0%)	42 (72.0%)	29 (81.0%)	0.700	0.540
History of C-section	148 (9.4%)	78 (15.2%)	31 (18.1%)	10 (17.2%)	12 (33.3%)	< 0.001	0.120
History of abortion^c^	403 (25.6%)	149 (29.1%)	56 (32.7%)	19 (32.8%)	16 (44.4%)	0.020	0.390
Smoker	21 (1.3%)	8 (1.6%)	3 (1.8%)	3 (5.2%)	1 (2.8%)	0.150	0.310
Pre-existing HTN	12 (0.8%)	6 (1.2%)	4 (2.3%)	6 (10.3%)	2 (5.6%)	< 0.001	0.031
Pre-existing DM^d^	13 (0.8%)	5.0 (1.0%)	4 (2.3%)	4 (6.9%)	1 (2.8%)	0.003	0.26

Baseline characteristics of the study population

p-Values are shaded grey

Abbreviations: ART, assisted reproductive technology; BMI, body mass index; C-section, cesarean section; HTN, hypertension; DM, diabetes mellitus

^a^Mean result (standard deviation)

^b^Median result (interquartile range)

^c^At least two abortions

^d^Diabetes mellitus type 1 and 2

Overall, approximately 11% of the population was obese. The mean age was higher in the reference group (p = 0.001) while being similar within the obese group. The use of ART as well as the gestational age were not significant in both comparisons. Normal weight mothers were significantly more often primigravida than overweight and obese mothers (p < 0.001). Overweight mothers were almost twice as often primigravida as obesity level III mothers (42.0% vs. 22.2%) The median number of gravidities was twice as high in the overweight and obese group (*p* < 0.001). Rates of dichorionic pregnancies were similar within all groups (78% vs. 76% vs. 79% vs. 72% vs. 81% respectively). History of cesarean sections and abortions as well as pre-existing diabetes mellitus type I or II were statistically significant in the comparison of normal weight mothers and the other groups (*p* < 0.001, *p* = 0.020 and *p* = 0.003 respectively). Rates of history of cesarean sections in the obesity level III group were almost three times as high as in the normal weight group and nearly doubled compared to obesity level I and II. In the comparison within the obese group, merely a pre-existing hypertension reached statistical significance (*p* = 0.031), which was also statistically significant in the comparison with the normal weight group (*p* < 0.001). Smoking during the pregnancy did not reach statistical significance, rates of smoking however were almost five times higher among women with obesity level II than normal weight or overweight mothers and mothers with obesity level I, as well as nearly twice as high as in obesity level III.

The following maternal and obstetric outcomes were analyzed with normal weighted mothers as reference group: gestational diabetes mellitus, preeclampsia of any severity, postpartum hemorrhage, fetal demise of at least one twin, and a five-minutes Apgar score below seven. The results of the crude analysis are shown in Tables 2 and 3.

**Table 2:** Crude logistic regression analysis of the maternal outcomes.

Exposure	GDM		PE		PPH	
	OR (95% CI)	*p*-Value	OR (95% CI)	*p*-Value	OR (95% CI)	*p*-Value
Overweight	1.48 (1.09–2.00)	0.011	1.24 (0.85–1.79)	0.265	0.97 (0.68–1.39)	0.859
Obesity level I	2.67 (1.79–3.97)	< 0.001	1.33 (0.76–2.34)	0.323	1.32 (0.79–2.19)	0.285
Obesity level II	3.26 (1.77–6.00)	< 0.001	1.02 (0.36–2.89)	0.963	0.38 (0.09–1.56)	0.179
Obesity level III	5.94 (2.98–11.86)	< 0.001	1.26 (0.38–4.17)	0.708	0.62 (0.15–2.61)	0.516

Results of the univariate logistic regression with normal weight as reference group

p-Values are shaded grey

Abbreviations: CI, confidence interval; GDM, gestational diabetes mellitus; OR, odds ratio; PE, preeclampsia; PPH, postpartum hemorrhage

**Table 3:** Crude logistic regression analysis of the neonatal outcomes.

Exposure	Fetal demise		5’-Apgar <7	
	OR (95% CI)	*p-Value*	OR (95% CI)	*p-Value*
Overweight	1.53 (0.93–2.52)	0.095	1.33 (0.83–2.12)	0.241
Obesity level I	1.33 (0.59–2.98)	0.493	1.70 (0.88–3.30)	0.115
Obesity level II	1.70 (0.51–5.61)	0.387	0.88 (0.21–3.71)	0.867
Obesity level III	1.83 (0.43–7.83)	0.416	1 (-)	-

Results of the univariate logistic regression with normal weight as reference group

p-Values are shaded grey

Abbreviations: CI, confidence interval; OR, odds ratio

Results of the crude analysis showed a statistically significant association between maternal weight and the risk for GDM (*p* = 0.011 for overweight, and *p* < 0.001 for each obesity level). Odds for gestational diabetes increased with overweight and every obesity level (1.48 vs. 2.67 vs. 3.26 vs. 5.94 respectively), mothers with obesity level III thus having a nearly six-fold risk higher risk for GDM than normal weight mothers. Preeclampsia and PPH were not significantly associated with maternal weight in the crude analysis, odds for PPH were even lowest in the obesity class II group. The analysis of the neonatal outcomes did not show statistically significant results either, odds for fetal demise were similar across all weight groups. Regarding the Apgar score, no score below seven after five minutes was recorded for mothers with obesity level III.

After adjusting all outcomes for covariates (Tables 4 and 5), gestational diabetes remained significantly more frequent among overweight and obese mothers (p = 0.015 and *p* < 0.001 for all obesity levels respectively). Odds ratios continued rising in parallel with obesity levels (2.79 vs. 4.05 vs. 6.40 respectively). Adjustment for covariates increased odds for preeclampsia as well as PPH, even though not significantly. Odds for preeclampsia remained the lowest among mothers with obesity level II, the highest odds however now being among obesity level III mothers, the latter therefore rather as expected. Odds for PPH persisted being the highest in the obesity level I group. Results of the neonatal outcomes did not alter in the adjusted analysis, odds being similar to the crude analysis.

**Table 4:** Adjusted analysis of the maternal outcomes.

Predictors	GDM		PE		PPH	
	aOR (95% CI)	*p*-Value	aOR (95% CI)	*p*-Value	aOR (95% CI)	*p*-Value
Exposure: overweight and obesity levels (reference: normal weight)						
Overweight	1.47 (1.08–1.99)	0.015	1.36 (0.93–1.98)	0.110	0.99 (0.69–1.43)	0.968
Obesity level I	2.79 (1.86–4.18)	< 0.001	1.47 (0.82–2.63)	0.191	1.41 (0.83–2.38)	0.200
Obesity level II	4.05 (2.14–7.65)	< 0.001	1.06 (0.36–3.14)	0.916	0.42 (0.10–1.77)	0.237
Obesity level III	6.40 (3.11–13.17)	< 0.001	1.56 (0.45–5.43)	0.483	0.78 (0.18–3.34)	0.737
Age (in years)	1.05 (1.02–1.08)	0.001	1.03 (0.10–1.07)	0.059	1.02 (0.98–1.05)	0.321
ART	1.06 (0.79–1.43)	0.690	1.07 (0.74–1.53)	0.728	1.39 (0.99–1.96)	0.057
Primipara	0.85 (0.58–1.26)	0.421	1.63 (0.89–2.99)	0.114	1.32 (0.80–2.17)	0.271
Gravidity	1.02 (0.87–1.19)	0.834	0.72 (0.54–0.96)	0.023	0.95 (0.76–1.18)	0.624
Dichorionicity	1.06 (0.77–1.46)	0.72	1.12 (0.74–1.70)	0.605	0.70 (0.48–1.00)	0.053
History of C-section	1.18 (0.80–1.74)	0.402	0.73 (0.35–1.50)	0.394	0.65 (0.36–1.20)	0.170
History of abortions^a^	1.04 (0.72–1.49)	0.847	2.59 (1.47–4.60)	0.001	1.42 (0.88–2.30)	0.154
Smoking	1.25 (0.46–3.40)	0.659	0.42 (0.05–3.35)	0.411	0.40 (0.54–2.98)	0.371
Pre-existing HTN	0.52 (0.15–1.83)	0.307	2.64 (1.01–6.89)	0.048	0.83 (0.19–3.65)	0.801
Pre-existing DM^b^	-	-	2.34 (0.75–7.30)	0.144	1.74 (0.50–6.09)	0.388
Gestational age (in weeks)						
24–27+6	-	-	-	-	0.18 (0.54–0.62)	0.006
28–31+6	-	-	-	-	0.78 (0.23–0.27)	< 0.001
32–36+6	-	-	-	-	0.18 (0.72–0.47)	< 0.001
≥ 37+0	-	-	-	-	0.19 (0.74–0.49)	0.001

Results of the adjusted multivariate logistic regression with normal weight as reference group

p-Values are shaded grey

Abbreviations: aOR, adjusted odds ratio; ART, assisted reproductive technology; CI, confidence interval; C-section, cesarean section; DM, diabetes mellitus; GDM, gestational diabetes mellitus; HTN, hypertension; PE, preeclampsia; PPH, postpartum hemorrhage

^a^At least two abortions

^b^Diabetes mellitus type 1 and 2

**Table 5:** Adjusted analysis of the neonatal outcomes.

Predictors	Fetal demise		5‘-Apgar <7	
	aOR (95% CI)	*p*-Value	aOR (95% CI)	*p*-Value
Exposure: overweight and obesity levels (reference: normal weight)				
Overweight	1.53 (0.91–2.59)	0.112	1.15 (0.68–1.94)	0.609
Obesity level I	1.45 (0.62–3.40)	0.389	1.63 (0.77–3.44)	0.200
Obesity level II	1.70 (0.48–6.05)	0.412	0.43 (0.09–2.04)	0.286
Obesity level III	2.40 (0.49–11.64)	0.277	1.00 (-)	-
Age (in years)	1.03 (0.98–1.08)	0.228	0.96 (0.91–1.00)	0.063
ART	1.29 (0.74–2.24)	0.375	1.36 (0.80–2.31)	0.250
Primipara	1.35 (0.62–2.91)	0.447	0.97 (0.48–1.96)	0.942
Gravidity	0.84 (0.60–1.19)	0.327	1.25 (0.94–1.66)	0.120
Dichorionicity	0.27 (0.16–0.45)	<0.001	0.69 (0.42–1.14)	0.151
History of C-section	0.72 (0.30–1.75)	0.469	0.64 (0.29–1.44)	0.283
History of abortions^a^	1.70 (0.82–3.56)	0.157	0.81 (0.42–1.59)	0.548
Smoking	0.87 (0.11–6.92)	0.891	1.88 (0.45–7.94)	0.388
Pre-existing HTN	1.00 (-)	-	1.23 (0.15–10.32)	0.846
Pre-existing DM^b^	1.73 (0.22–13.65)	0.601	2.26 (0.48–10.64)	0.304
Gestational age (in weeks)				
24–27+6	0.88 (0.28–2.81)	0.831	0.79 (0.28–2.23)	0.657
28–31+6	0.26 (0.80–0.86)	0.027	0.34 (0.13–0.93)	0.036
32–36+6	0.12 (0.04–0.34)	< 0.001	0.05 (0.19–0.14)	< 0.001
≥ 37+0	0.13 (0.04–0.38)	< 0.001	0.02 (0.33–12.44)	< 0.001

Results of the adjusted multivariate logistic regression with normal weight as reference group

p-Values are shaded grey

Abbreviations: aOR, adjusted odds ratio; ART, assisted reproductive technology; CI, confidence interval; C-section, cesarean section; DM, diabetes mellitus; HTN, hypertension

^a^At least two abortions

^b^Diabetes mellitus type 1 and 2

After adjustment for covariates, the influence of the maternal BMI as a continuous variable on all outcomes was analyzed (Table 6). GDM remained statistically significant (*p* < 0.001). Furthermore, a significant influence on the risk for preeclampsia could be shown as well (*p* = 0.028), thus confirming its increasing risk with rising maternal pre-pregnancy BMI.

**Table 6:** BMI as a continuous variable.

Exposure	Outcome	aOR (95% CI)	*p*-Value
BMI as a continuous variable	GDM	1.09 (1.07–1.12)	< 0.001
	PE	1.04 (1.00–1.07)	0.028
	PPH	1.01 (0.98–1.04)	0.614
	Fetal demise	1.04 (1.00–1.09)	0.077
	5’-Apgar <7	0.99 (0.94–1.03)	0.598

Effect of the maternal BMI as a continuous variable on all outcomes

p-Values are shaded grey

The covariates of each adjusted regression were similar to Tables 4 and 5

Abbreviations: aOR, adjusted odds ratio; BMI, body mass index; CI, confidence interval; GDM, gestational diabetes mellitus; PE, preeclampsia; PPH, postpartum hemorrhage

## Discussion

Twin as well as multiple pregnancies are at a higher risk for gestational diabetes mellitus [15–17]. According to Roach et al. [18], every additional fetus elevates the risk for GDM by 1.8 times. Additionally, obesity significantly increases the risk for gestational diabetes in both singleton and twin pregnancies [1, 8, 15, 16, 19, 20], possibly due to an increased weight gain with twins and higher blood pressures both before and during pregnancy [15]. Consistent with these studies, data of this analysis also showed a significant association between GDM and overweight or obesity, with increasing odds ratios with each obesity level. Overweight mothers had a one-and-a-half-fold higher risk of suffering from GDM compared to normal weight mothers, this risk increasing up until a sixfold higher risk for obesity level III mothers. Furthermore, the BMI showed a significant influence on the risk for gestational diabetes, highlighting the even greater risk for GDM among pregnant mothers with a BMI ≥ 40 kg/m^2^. With globally increasing rates of gestational diabetes mellitus, correlating to the rising numbers of twin pregnancies [21], and GDM being one of the most frequent pregnancy complications [1], prevention and information on the risks of gestational diabetes should be extended.

Many studies showed that twin pregnancies per se increase the risk of preeclampsia, and that obesity, regardless of the number of fetuses, favors preeclampsia as well [22, 23]. According to Young et al. [24], the risk of preeclampsia grows with increasing pre-pregnancy BMI. Aviram et al. [22] described a two-fold higher risk of preeclampsia in twin pregnancies. Lučovnik et al. [25] confirmed that the combination of both factors, i.e. obesity and twin pregnancies, further promotes preeclampsia. It has furthermore been described that the maternal BMI presented the most important risk factor for developing preeclampsia, a BMI ≥ 30 kg/m^2^ significantly increasing this risk. In addition, there was a direct correlation as a dose-response-relationship between the BMI and preeclampsia, implicating that obese mothers carried a significantly higher risk than overweight mothers [25]. In this study cohort, preeclampsia did not show significant results in the crude and adjusted analyses. However, the maternal BMI did have a significant effect on preeclampsia, confirming the results of the studies mentioned above. Some studies described an association between GDM and preeclampsia in twin pregnancies [25–29]. A possible explanation for this correlation might be found in a similar pathophysiology, such as an increased insulin resistance in preeclamptic mothers [30, 31], which also plays a role in the pathogenesis of gestational diabetes and is favored by obesity [32]. Interactions between preeclampsia and GDM in twin pregnancies could therefore form the basis for further research.

PPH is the most important reason for pregnancy-related morbidity, 70% of all PPH being caused by uterine atony or inadequate uterine contraction [33]. Maternal obesity and multiple pregnancies are considered being important risk factors for PPH, multifetal gestations possibly leading into uterine overdistension with the consequent risk of PPH [33, 34]. The influence of the mode of delivery on the risk for PPH in twin pregnancies still lacks research. Suzuki et al. [35] described an 18-fold higher risk for PPH in twins with vaginal delivery. However, the impact of cesarean deliveries of twins has not been studied thoroughly. In singleton pregnancies, data are inconsistent, some describing an up to 19% higher risk of PPH after vaginal deliveries, especially in overweight and obese mothers [36], others again reporting a two- to threefold higher risk after cesarean sections, however with a dose-dependent relationship between maternal BMI and the risk as well [37]. Albeit not reaching significance, results of this study showed a nearly one-and-a-half fold higher risk for PPH in obesity level I mothers, with however decreasing odds in obesity level II and III mothers. Disparities between these findings and results of the studies mentioned above might be caused by a high rate of cesarean deliveries (approximately 72%) in this cohort, the latter possibly allowing deductions on lower rates of PPH after cesarean sections in twins and thus supporting the results of Suzuki et al. [35] Nevertheless, a study with greater statistical power is required for more reliable statements on the risk for PPH in twin deliveries in general, as well as in dependence of the mode of delivery.

In both singleton and twin pregnancies, obesity was shown to increase the risk for adverse fetal outcomes [3, 9, 38–40]. In singleton pregnancies, a 50% higher likelihood for stillbirth among obese mothers compared to normal weight mothers was described [41]. Furthermore, the highest risk is present among mothers with obesity level III, having 100% higher odds for stillbirth compared to normal weight mothers [41]. Overall, obesity and overweight were shown to elevate the risk of fetal death, regardless of the number of fetuses [42]. Although not statistically significant, results of this study showed increasing odds for fetal demise with rising obesity level, mothers with obesity level III having a more than twofold higher risk compared to mothers of normal weight.

Studies on the impact of maternal weight on the Apgar score in twins show conflicting results. Findings of Bautista-Castaño et al. [39] showed no association between maternal obesity of any severity and low Apgar scores after five minutes. On the other hand, other studies have shown significantly higher rates of low Apgar scores with increasing pre-pregnancy BMI in both singleton and twin pregnancies [1, 43, 44]. The latter could not be confirmed by this study. Contrary to expectations, the odds of a 5’-Apgar score below seven were even lower in mothers in obesity level II than in mothers in obesity level I; surprisingly in the obesity level III group, no case of a 5’-Apgar score < 7 was documented. Further studies are therefore needed to draw reliable conclusions on this topic, also taking into consideration the impact of the mode of delivery, especially cesarean sections, the latter influencing outcomes such as fetal respiratory distress among others [45].

This study contributes to the still small range of studies on the effects of obesity on fetomaternal outcomes in twin pregnancies. Most studies are conducted analyzing the impact of overweight and obesity in general. In this study, obesity has been explored further by subdividing this weight group into the three obesity levels as defined by the World Health Organization, allowing a more detailed view on their impact on fetomaternal outcomes in twin pregnancies. The limitations include the retrospective nature of the study and the comparatively small study population, which might explain why some of the results were not significant in contrast to findings of other similar studies. Furthermore, given that Buergerhospital Frankfurt is a tertiary referral center with the highest birth rate of all hospitals in Germany and a focus on multiple pregnancies and their complications, the rates of pregnancy and postpartum complications may be biased due to the more intensive monitoring and, if necessary, early treatment of complications in high-risk pregnancies. Supplementary studies with greater statistical power should be conducted, especially to further elucidate the impacts of covariates such as mode of delivery on PPH or the Apgar score, as well as to investigate the influence of interactions between gestational diabetes and preeclampsia on fetomaternal outcomes in general, given that a similar pathophysiology has already been described.

## Conclusion

With twin pregnancies already being at high risk for adverse outcomes regardless of the maternal pre-pregnancy weight [3], this study supports findings of other studies on the additional impact of obesity on both mother and twins. However, this study also shows a lower rate of complications in contrast to some studies mentioned above, implicating the need for further studies.

With increasing incidences of overweight and obesity worldwide [6], the associated risks for twin pregnancies need to be addressed more to create a foundation for better understanding and enhanced prevention of overweight and obesity both in general and especially in regard to pregnancies.

## Supporting information

S1 Dataset (XLSX)

S1 File (DOCX)
