# Supplementary material for: The association between maternal obesity and fetomaternal outcomes in twin pregnancies
Source: PLoS One. 2024 Jul 10;19(7):e0306877. doi: 10.1371/journal.pone.0306877 (PMC11236179; doi:10.1371/journal.pone.0306877)
Supplement: S1 File — (DOCX) [file pone.0306877.s002.docx]

# Full results of the performed regression analyses:

Table 2. Crude logistic regression analysis of the maternal outcomes

| Exposure | GDM | | PE | | PPH | |
| --- | --- | --- | --- | --- | --- | --- |
|  | OR  (95% CI) | *p*-Value | OR  (95% CI) | *p*-Value | OR  (95% CI) | *p*-Value |
| Overweight | 1.48  (1.09-2.00) | 0.011 | 1.24  (0.85-1.79) | 0.265 | 0.97  (0.68-1.39) | 0.859 |
| Obesity  level I | 2.67  (1.79-3.97) | < 0.001 | 1.33  (0.76-2.34) | 0.323 | 1.32  (0.79-2.19) | 0.285 |
| Obesity  level II | 3.26  (1.77-6.00) | < 0.001 | 1.02  (0.36-2.89) | 0.963 | 0.38  (0.09-1.56) | 0.179 |
| Obesity  level III | 5.94  (2.98-11.86) | < 0.001 | 1.26  (0.38-4.17) | 0.708 | 0.62  (0.15-2.61) | 0.516 |

Results of the univariate logistic regression with normal weight as reference group

p-Values are shaded grey

Abbreviations: CI, confidence interval; GDM, gestational diabetes mellitus; OR, odds ratio; PE, preeclampsia; PPH, postpartum hemorrhage

Table 3. Crude logistic regression analysis of the neonatal outcomes

| Exposure | Fetal demise | | 5’-Apgar <7 | |
| --- | --- | --- | --- | --- |
|  | OR  (95% CI) | *p-Value* | OR  (95% CI) | *p-Value* |
| Overweight | 1.53  (0.93-2.52) | 0.095 | 1.33  (0.83-2.12) | 0.241 |
| Obesity  level I | 1.33  (0.59-2.98) | 0.493 | 1.70  (0.88-3.30) | 0.115 |
| Obesity  level II | 1.70  (0.51-5.61) | 0.387 | 0.88  (0.21-3.71) | 0.867 |
| Obesity  level III | 1.83  (0.43-7.83) | 0.416 | 1  (-) | - |

Results of the univariate logistic regression with normal weight as reference group

p-Values are shaded grey

Abbreviations: CI, confidence interval; OR, odds ratio

Table 4. Adjusted analysis of the maternal outcomes

| Predictors | GDM | | |  | PE | | PPH | | |
| --- | --- | --- | --- | --- | --- | --- | --- | --- | --- |
|  | aOR  (95% CI) | SE | *p*-Value | aOR  (95% CI) | SE | *p*-Value | aOR  (95% CI) | SE | *p*-Value |
| Exposure: overweight and obesity levels (reference: normal weight) |  |  |  |  |  |  |  |  |  |
| Overweight | 1.47  (1.08-1.99) | 0.23 | 0.015 | 1.36  (0.93-1.98) | 0.26 | 0.110 | 0.99  (0.69-1.43) | 0.18 | 0.968 |
| Obesity  level I | 2.79  (1.86-4.18) | 0.58 | < 0.001 | 1.47  (0.82-2.63) | 0.44 | 0.191 | 1.41  (0.83-2.38) | 0.38 | 0.200 |
| Obesity  level II | 4.05  (2.14-7.65) | 1.31 | < 0.001 | 1.06  (0.36-3.14) | 0.59 | 0.916 | 0.42  (0.10-1.77) | 0.31 | 0.237 |
| Obesity  level III | 6.40  (3.11-13.17) | 2.36 | < 0.001 | 1.56  (0.45-5.43) | 0.99 | 0.483 | 0.78  (0.18-3.34) | 0.58 | 0.737 |
| Age (in years) | 1.05  (1.02-1.08) | 0.15 | 0.001 | 1.03  (0.10-1.07) | 0.12 | 0.059 | 1.02  (0.98-1.05) | 0.02 | 0.321 |
| ART | 1.06  (0.79-1.43) | 0.16 | 0.690 | 1.07  (0.74-1.53) | 0.20 | 0.728 | 1.39  (0.99-1.96) | 0.24 | 0.057 |
| Primipara | 0.85  (0.58-1.26) | 0.50 | 0.421 | 1.63  (0.89-2.99) | 0.50 | 0.114 | 1.32  (0.80-2.17) | 0.33 | 0.271 |
| Gravidity | 1.02  (0.87-1.19) | 0.10 | 0.834 | 0.72  (0.54-0.96) | 0.11 | 0.023 | 0.95  (0.76-1.18) | 0.11 | 0.624 |
| Dichorionicity | 1.06  (0.77-1.46) | 0.24 | 0.72 | 1.12  (0.74-1.70) | 0.24 | 0.605 | 0.70  (0.48-1.00) | 0.13 | 0.053 |
| History of  C-section | 1.18  (0.80-1.74) | 0.27 | 0.402 | 0.73  (0.35-1.50) | 0.27 | 0.394 | 0.65  (0.36-1.20) | 0.20 | 0.170 |
| History of abortions^a^ | 1.04  (0.72-1.49) | 0.76 | 0.847 | 2.59  (1.47-4.60) | 0.76 | 0.001 | 1.42  (0.88-2.30) | 0.35 | 0.154 |
| Smoking | 1.25  (0.46-3.40) | 0.41 | 0.659 | 0.42  (0.05-3.35) | 0.44 | 0.411 | 0.40  (0.54-2.98) | 0.41 | 0.371 |
| Pre-existing HTN | 0.52  (0.15-1.83) | 1.14 | 0.307 | 2.64  (1.01-6.89) | 1.29 | 0.048 | 0.83  (0.19-3.65) | 0.63 | 0.801 |
| Pre-existing DM^b^ | - | 1.26 | - | 2.34  (0.75-7.30) | 1.36 | 0.144 | 1.74  (0.50-6.09) | 1.11 | 0.388 |
| Gestational age (in weeks) |  |  |  |  |  |  |  |  |  |
| 24 – 27+6 | - | - | - | - | - | - | 0.18  (0.54-0.62) | 0.11 | 0.006 |
| 28 – 31+6 | - | - | - | - | - | - | 0.78  (0.23-0.27) | 0.05 | < 0.001 |
| 32 – 36+6 | - | - | - | - | - | - | 0.18  (0.72-0.47) | 0.09 | < 0.001 |
| ≥ 37+0 | - | - | - | - | - | - | 0.19  (0.74-0.49) | 0.09 | 0.001 |

Results of the adjusted multivariate logistic regression with normal weight as reference group

p-Values are shaded grey

Abbreviations: aOR, adjusted odds ratio; ART, assisted reproductive technology; CI, confidence interval; C-section, cesarean section; DM, diabetes mellitus; GDM, gestational diabetes mellitus; HTN, hypertension; PE, preeclampsia; PPH, postpartum hemorrhage; SE, standard error

^a^At least two abortions

^b^Diabetes mellitus type 1 and 2

Table 5. Adjusted analysis of the neonatal outcomes

| Predictors | Fetal demise | | | 5‘-Apgar <7 | | |
| --- | --- | --- | --- | --- | --- | --- |
|  | aOR  (95% CI) | SE | *p*-Value | aOR  (95% CI) | SE | *p*-Value |
| Exposure: overweight and obesity levels (reference: normal weight) |  |  |  |  |  |  |
| Overweight | 1.53  (0.91-2.59) | 0.41 | 0.112 | 1.15  (0.68-1.94) | 0.31 | 0.609 |
| Obesity  level I | 1.45  (0.62-3.40) | 0.63 | 0.389 | 1.63  (0.77-3.44) | 0.62 | 0.200 |
| Obesity  level II | 1.70  (0.48-6.05) | 1.10 | 0.412 | 0.43  (0.09-2.04) | 0.34 | 0.286 |
| Obesity  level III | 2.40  (0.49-11.64) | 1.93 | 0.277 | 1.00  (-) | - | - |
| Age (in years) | 1.03  (0.98-1.08) | 0.26 | 0.228 | 0.96  (0.91-1.00) | 0.02 | 0.063 |
| ART | 1.29  (0.74-2.24) | 0.36 | 0.375 | 1.36  (0.80-2.31) | 0.37 | 0.250 |
| Primipara | 1.35  (0.62-2.91) | 0.53 | 0.447 | 0.97  (0.48-1.96) | 0.35 | 0.942 |
| Gravidity | 0.84  (0.60-1.19) | 1.15 | 0.327 | 1.25  (0.94-1.66) | 0.18 | 0.120 |
| Dichorionicity | 0.27  (0.16-0.45) | 0.07 | <0.001 | 0.69  (0.42-1.14) | 0.18 | 0.151 |
| History of  C-section | 0.72  (0.30-1.75) | 0.33 | 0.469 | 0.64  (0.29-1.44) | 0.26 | 0.283 |
| History of abortions^a^ | 1.70  (0.82-3.56) | 0.64 | 0.157 | 0.81  (0.42-1.59) | 0.28 | 0.548 |
| Smoking | 0.87  (0.11-6.92) | 0.92 | 0.891 | 1.88  (0.45-7.94) | 1.38 | 0.388 |
| Pre-existing HTN | 1.00  (-) | - | - | 1.23  (0.15-10.32) | 1.34 | 0.846 |
| Pre-existing DM^b^ | 1.73  (0.22-13.65) | 1.83 | 0.601 | 2.26  (0.48-10.64) | 1.79 | 0.304 |
| Gestational age (in weeks) |  |  |  |  |  |  |
| 24 – 27+6 | 0.88  (0.28-2.81) | 0.52 | 0.831 | 0.79  (0.28-2.23) | 0.42 | 0.657 |
| 28 – 31+6 | 0.26  (0.80-0.86) | 1.16 | 0.027 | 0.34  (0.13-0.93) | 0.17 | 0.036 |
| 32 – 36+6 | 0.12  (0.04-0.34) | 0.63 | < 0.001 | 0.05  (0.19-0.14) | 0.03 | < 0.001 |
| ≥ 37+0 | 0.13  (0.04-0.38) | 0.07 | < 0.001 | 0.02  (0.33-12.44) | 0.01 | < 0.001 |

Results of the adjusted multivariate logistic regression with normal weight as reference group

p-Values are shaded grey

Abbreviations: aOR, adjusted odds ratio; ART, assisted reproductive technology; CI, confidence interval; C-section, cesarean section; DM, diabetes mellitus; HTN, hypertension; SE, standard error

^a^At least two abortions

^b^Diabetes mellitus type 1 and 2

Table 6. BMI as a continuous variable

| Exposure | Outcome | aOR  (95% CI) | *p*-Value |
| --- | --- | --- | --- |
| BMI as a  continuous  variable | GDM | 1.09  (1.07-1.12) | < 0.001 |
|  | PE | 1.04  (1.00-1.07) | 0.028 |
|  | PPH | 1.01  (0.98-1.04) | 0.614 |
|  | Fetal demise | 1.04  (1.00-1.09) | 0.077 |
|  | 5’-Apgar <7 | 0.99  (0.94-1.03) | 0.598 |

Effect of the maternal BMI as a continuous variable on all outcomes

p-Values are shaded grey

The covariates of each adjusted regression were similar to tables 4 and 5

Abbreviations: aOR, adjusted odds ratio; BMI, body mass index; CI, confidence interval; GDM, gestational diabetes mellitus; PE, preeclampsia; PPH, postpartum hemorrhage; SE, standard error

Gestational diabetes mellitus and preeclampsia being the only significant results in the adjusted analysis, the measures of goodness of fit were estimated for these outcomes only.

Gestational diabetes mellitus:

- Goodness-of-fit test: 0.104
- Akaike information criterion (AIC): 1699.1
- Bayesian information criterion (BIC): 1779.6

Preeclampsia:

- Goodness-of-fit test: 0.695
- Akaike information criterion (AIC): 1207.4
- Bayesian information criterion (BIC): 1293.8
